# Functional Characterization of Two M42 Aminopeptidases Erroneously Annotated as Cellulases

**DOI:** 10.1371/journal.pone.0050639

**Published:** 2012-11-30

**Authors:** Raphaël Dutoit, Nathalie Brandt, Christianne Legrain, Cédric Bauvois

**Affiliations:** Institut de Recherches Microbiologiques JM Wiame, Brussels, Belgium; University of Canterbury, New Zealand

## Abstract

Several aminopeptidases of the M42 family have been described as tetrahedral-shaped dodecameric (TET) aminopeptidases. A current hypothesis suggests that these enzymes are involved, along with the tricorn peptidase, in degrading peptides produced by the proteasome. Yet the M42 family remains ill defined, as some members have been annotated as cellulases because of their homology with CelM, formerly described as an endoglucanase of *Clostridium thermocellum*. Here we describe the catalytic functions and substrate profiles CelM and of TmPep1050, the latter having been annotated as an endoglucanase of *Thermotoga maritima*. Both enzymes were shown to catalyze hydrolysis of nonpolar aliphatic L-amino acid-pNA substrates, the L-leucine derivative appearing as the best substrate. No significant endoglucanase activity was measured, either for TmPep1050 or CelM. Addition of cobalt ions enhanced the activity of both enzymes significantly, while both the chelating agent EDTA and bestatin, a specific inhibitor of metalloaminopeptidases, proved inhibitory. Our results strongly suggest that one should avoid annotating members of the M42 aminopeptidase family as cellulases. In an updated assessment of the distribution of M42 aminopeptidases, we found TET aminopeptidases to be distributed widely amongst archaea and bacteria. We additionally observed that several phyla lack both TET and tricorn. This suggests that other complexes may act downstream from the proteasome.

## Introduction

In prokaryotic cells, protein degradation is a key mechanism in the quality control of proteins, homeostasis, regulation of the cell cycle, and responses to environmental stresses [1]–[3]. In archaea and actinomycetes, proteolysis is carried out by the 20S proteasome [4], [5], while in other prokaryotes, several protease complexes degrade proteins. These complexes are functionally related to the core particle of the 20S proteasome [6], [7]. The 20S proteasome and the protease complexes generate peptides 3 to 25 amino acids in length [8] that need to be processed further to amino acids. Degradation of these peptides is catalyzed by proteasome-unrelated amino- and carboxypeptidases. Over the last 15 years, two large proteolytic complexes have been identified: the tricorn protease (TRI) and the tetrahedral-shaped oligomeric aminopeptidase (TET). These complexes are thought to complete the degradation of peptides in prokaryotes, each organism being assumed to possess either TRI or TET [9]. The first characterized TRI, isolated from the archaeon *Thermoplasma acidophilum* as a 720-kDa hexameric protein displaying carboxypeptidase activity, was classified as an S41 serine endopeptidase [10], [11]. *In vivo*, TRI organizes into 14.6-MDa icosahedral capsids containing 20 hexamers [12]. Furthermore, three monomeric aminopeptidases (F1, F2, and F3) are associated with TRI, broadening its substrate specificity [10], [13], [14]. TRI is not widely found in prokaryotic organisms, having been identified in only two archaea and some bacteria such as *Streptomyces coelicolor* [15], [16]. TET, on the other hand, were discovered recently in both the archaea *Haloarcula marismortui* and *Pyrococcus horikoshii* [17]–[19] and the Gram+ bacterium *Streptococcus pneumoniae* [20]. They are dodecameric aminopeptidases belonging to the M42 dinuclear aminopeptidase family [21], able to hydrolyze peptides up to 40 residues long [19].

There is confusion, however, as regards the functional classification of the M42 aminopeptidases. Two different views exist. On the one hand, the MEROPS (http://merops.sanger.ac.uk/) definition outlines a sub-family of non-peptidase homologs sharing homology with CelM, an endoglucanase of *Clostridium thermocellum* [22]. On the other hand, the NCBI Conserved Domain Database (CDD) defines the M42 aminopeptidase family (accession number cd05638) as comprising aminopeptidases (including TET), endoglucanases, and proteins of the Frv operon, described to be involved in the biosynthesis and the degradation of polysaccharides [23]. Recently, despite the earlier attribution of CelM, several of its homologs (from *P. horikoshii* [24], *Symbiobacterium thermophilum* [25], and *Cytophaga*-like bacteria [26]) have recently been characterized as aminopeptidases.

To avoid misannotating the members of this protein family as endoglucanases and in order to redefine the M42 aminopeptidase family, it is necessary to characterize the enzymatic activities of CelM. Here we have characterized the activities of both CelM and the *Thermotoga maritima* protein TmPep1050 (pdb code 3ISX), which is structurally related to the M42 aminopeptidases but annotated as an endoglucanase. As our results suggest that CelM-like proteins are not cellulases but aminopeptidases, we propose an updated distribution of M42 aminopeptidases within the Archaea and the Bacteria. Our results highlight the difficulty of deducing the catalytic activity of a protein from its protein sequence on the basis of the biochemical characterization of only one enzyme. Such an approach may frequently lead to annotation errors in sequence databases.

**Figure 1:**
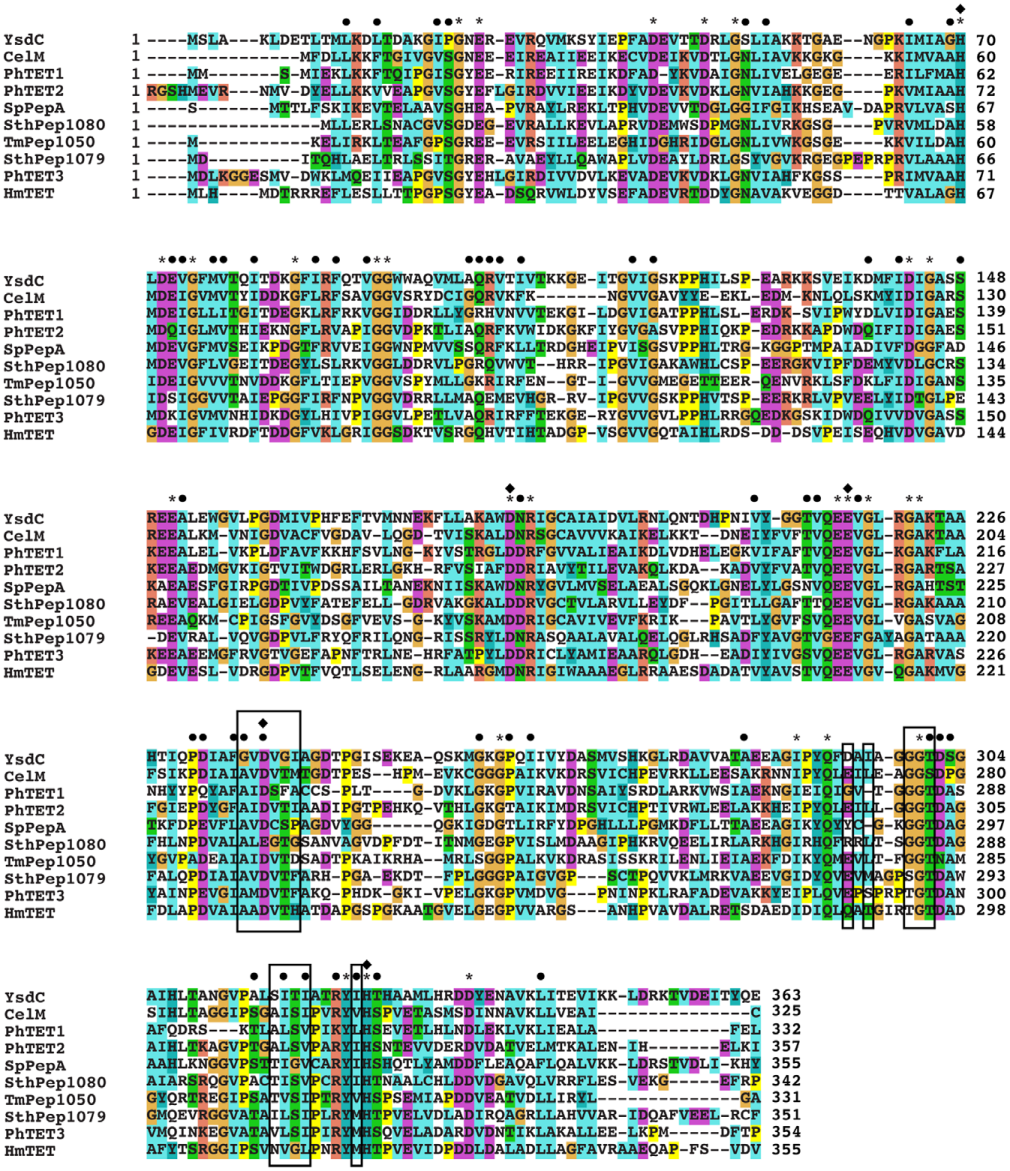
Sequence alignment of TmPep1050 and CelM vs. the characterized M42 aminopeptidases. Characterized M42 aminopeptidases used for the multiple alignment: PhTET1, PhTET2, and PhTET3 from *P. horikoshii* [17], [18], [34], HmTET from *Haloarcula marismortui* [19], SpPepA from *S. pneumoniae* [20], SthPep1079 and SthPep1080 from *Symbiobacterium thermophilum* [25], and YsdC (pdb code 1VHE) from *Bacillus subtilis* [45]. * and • indicate, respectively, conserved amino acids and residues with similar properties. ♦ indicates amino acids involved in metal ion binding. Amino acids constituting the S1 pocket defined from structural studies of SpPepA and PhTET2 are highlighted in black boxes.

## Materials and Methods

### Plasmids, Strains, Growth Media, and Reagents

Plasmid TmCD00089984 was designed by the Joint Center for Structural Genomics (JCSG) and carries the TM1050 ORF of *T. maritima* coding for the TmPep1050 protein under the control of the arabinose-inducible *araBAD* promoter. Plasmid pET28b+CelM, kindly provided by Pr. David Wilson (Cornell University, Ithaca, NY), allows production, via an IPTG-inducible T7 system (Novagen), of *C. thermocellum* CelM bearing a C-terminal hexahistidine tag. Plasmids were maintained in *E. coli* DH5α (Invitrogen). *E. coli* MC1061 [27] and BL21(DE3) (Novagen) were used for expression, and were transformed with TmCD00089984 and pET28b+CelM respectively. The strains were grown in LB medium supplemented with 100 µg/mL ampicillin (in the case of MC1061 harboring TmCD00089984) or 50 µg/mL kanamycin (in the case of BL21 (DE3) harboring pET28b+CelM). Amino acid-p-nitroanilide (pNA) substrates were purchased from Bachem AG. Z-peptides were purchased from Sigma-Aldrich NV/SA.

**Table 1:** Characterization of the endoglucanase activity of TmPep1050 and CelM.

Substrates	*A. niger* cellulase sp act (µmol min^−1^ U^−1^)	CelM sp act (µmol min^−1^mg^−1^)	TmPep1050 sp act (µmol min^−1^ mg^−1^)
Cellobiose	108.8±8.6	<0.1	<0.1
Carboxymethyl cellulose	359.0±15.5	<0.1	<0.1
Cellulose (Whatman n°1 filter paper)	67.6±1.7	<0.05	<0.05

Activities are expressed in µmol reduced sugar produced by substrate hydrolysis and were estimated by the DNS method. SEM is given for each value.

### Production and Purification of Recombinant Enzymes

Cells were grown in 1 L LB broth at 37°C to OD_660nm_ = 0.6 (about 6×10^8^ cells mL^−1^). The cultures were cooled to 18°C and induced by adding 0.2 g/L arabinose or 1 mM IPTG depending on the plasmid. Induction was carried out for 14 hours at 18°C. Cells were harvested by centrifugation at 5,500× g (Sorvall RC-6, SLA1500 rotor), washed with 0.9% NaCl, and frozen at -80°C. Prior to protein extraction, the cells were thawed in 40 mL of 50 mM Tris, 300 mM NaCl buffer pH 7.3 supplemented with Complete EDTA-free Protease Inhibitor (Roche Applied Science) and 250 U benzonase (Merck biosciences). Cells were disrupted by sonication (Ultrasonic Inc., W-225R) and insoluble particles were pelleted by centrifugation (30 min at 17,500× g, Sorvall RC-6, SS34 rotor). As both *T. maritima* and *C. thermocellum* are thermophilic bacteria, the clarified cell extracts were heated at 60°C for 15 min and coagulated proteins were removed by centrifugation (30 min at 17,500× g, Sorvall RC-6, SS34 rotor). The supernatants were used directly for purification. Recombinant CelM and TmPep1050 were purified by ion metal affinity chromatography (IMAC) on Ni-nitrilotriacetic acid agarose resin (Qiagen) in 50 mM Tris, 300 mM NaCl buffer pH 7.3. Elution was performed in three steps with increasing concentrations of imidazole (100, 300, and 500 mM). Fractions corresponding to the elution peak at 300 mM imidazole were pooled and applied to a Superdex 200 (GE Healthcare, 16/70 column) gel filtration resin in 50 mM Tris, 300 mM NaCl buffer pH 7.3. Fractions containing the protein of interest were pooled and concentrated on a Vivaspin 15R 30 kDa (Sartorius). The presence and purity of the recombinant enzymes were checked throughout the purification procedure by SDS-PAGE. For purification of CelM, all buffers contained glutathione at 1 mM as a stabilizing agent and the purified protein was dialyzed against 65 mM HEPES, 520 mM sodium tartrate, 25% glycerol buffer pH 7.5.

**Table 2:** Characterization of the aminopeptidase activity of TmPep1050 and CelM.

Substrates	TmPep1050 sp act	CelM sp act
L-Lys-pNA	0.6±0.1	n.d.
L-Glu-pNA	n.d.	n.d.
L-Pro-pNA	n.d.	n.d.
L-His-pNA	n.d.	n.d.
L-Phe-pNA	n.d.	n.d.
L-Gly-pNA	n.d.	n.d.
L-Ala-pNA	2.4±0.2	2.1±0.1
L-Val-pNA	14.3±1.8	8.4±0.016
L-Leu-pNA	188.9±8.1	291.7±12.6
L-Ile-pNA	76.4±10.0	53.1±13.1
L-Met-pNA	27.5±0.7	14.6±1.5
D-Leu-pNA	n.d.	n.d.
L-Leu-pNA with EDTA 0.7 mM	3.2±0.6	2.4±0.9

Specific activities (sp act) are expressed in µmol of p-nitroaniline produced by hydrolysis of the amino acid-pNA derivative per minute and per µmol of enzyme. SEM is given for each value. Enzymatic reactions were run as described in the Materials & Methods section. n.d. not detectable, measured activity below 0.5 µmol min^−1^ µmol^−1^.

### Enzymatic Assays

Endoglucanase (EC 3.2.1.4) activity was characterized by measuring the degradation of carboxymethyl cellulose (CMC), cellobiose, and Whatman N°1 filter paper as described previously [28]. Reducing sugars were quantified by the dinitrosalicylic acid (DNS) method. A cellulase from *Aspergillus niger* (Sigma-Aldrich, C-1184) was used as a positive control with 1.24 unit of cellulase in each assay.

**Figure 2:**
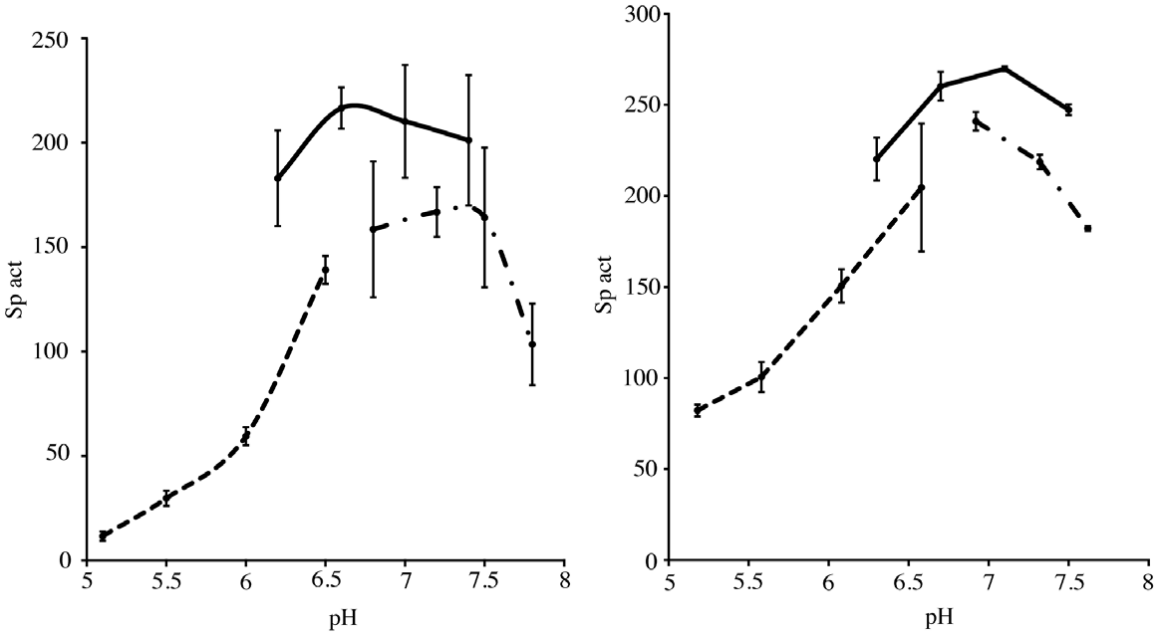
Influence of pH on the LAP activity of (A) TmPep1050 and (B) CelM. Curves of activity vs. pH in the MES **(**dashed line), MOPS (solid line), and HEPES (dash-dotted line) buffers. Specific activities (sp act) are expressed in µmol of p-nitroaniline produced by hydrolysis of the amino acid-pNA derivative per minute and per µmol of enzyme.

Carboxypeptidase (EC 3.4.17.1) activity assays were performed in 50 mM MOPS buffer pH 7.0 at 60°C with 1 mM *N*-terminally blocked peptide (Z-Gly-Tyr, Z-Ala-Glu, and Z-Gly-Gly-Leu). 25 µg recombinant enzyme was used in each assay. Free amino acids were quantified with the hydrindantin-ninhydrin reagent [29]. A dinuclear carboxypeptidase from *Sulfolobus solfataricus*, SsoCP2 (unpublished data), was used as positive control with 0.5 µg SsoCP2 in each assay.

**Figure 3:**
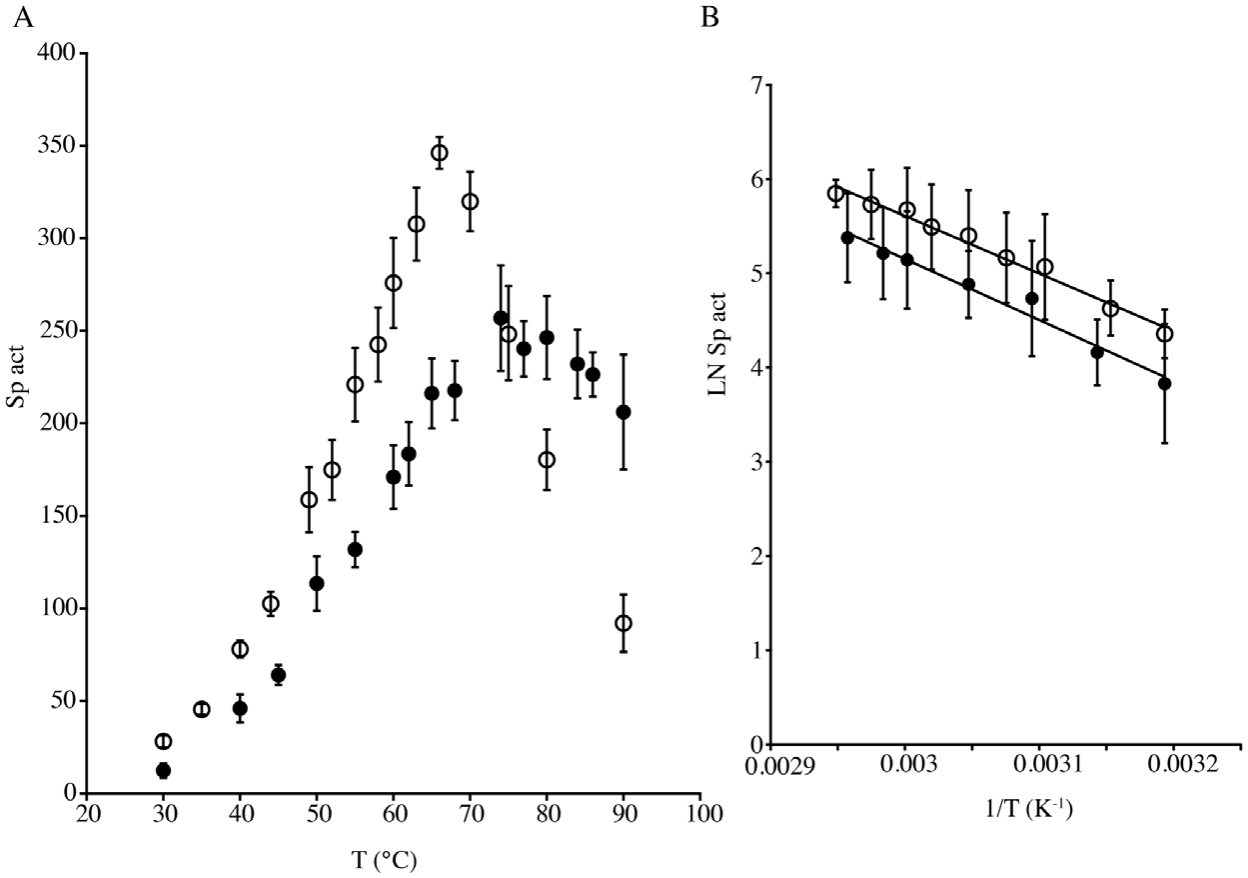
Influence of temperature on the LAP activity of TmPep1050 (closed circles) and CelM (open circles). (A) Activity-vs.-temperature plot; (B) Plot showing the logarithm of the activity vs. the inverse of the temperature. Specific activities (sp act) are expressed in µmol of p-nitroaniline produced by hydrolysis of the amino acid-pNA derivative per minute and per µmol of enzyme. Trend lines were calculated by linear regression (R^2^ = 0.9735 for TmPep1050, and R^2^ = 0.9853 for CelM).

Aminopeptidase (EC 3.4.11.1) activity was assayed as previously described [30], with the following modifications: reactions were run in 50 mM MOPS buffer pH 7.0 containing 10% methanol. For functional characterization of TmPep1050 and CelM, aminopeptidase assays were carried out at 60°C with 8 µg recombinant enzyme and the appropriate amino acid-p-NA substrate. All substrates were used at 2.5 mM, except L-Gly-pNA (1.25 mM), L-Phe-pNA (0.75 mM), L-His-pNA, and L-Glu-pNA (0.5 mM). Activity assays in the absence of Co^2+^ and in the presence of 0.7 mM EDTA were performed with 2.5 mM L-Leu-pNA. Metal ion, pH, and temperature dependence studies were carried out at 2.5 mM L-Leu-pNA. For the determination of kinetic parameters, assays were performed at enzyme concentrations ranging from 2 to 16 µg/mL and under optimal conditions as regards cobalt ion concentration, pH, and temperature: 1 mM Co^2+^, pH 7.0, and 60°C for CelM; 0.1 mM Co^2+^, pH 6.6, 75°C for TmPep1050. Kinetic parameters (k_cat_, K_m_ and k_cat_/K_m_) were determined from the initial reaction rates, using Lineweaver-Burk linearization of the Michaelis-Menten equation. The apparent inhibition constant (K_i_) for bestatin was determined by plotting the inverse of the specific activity vs. the bestatin concentration. Activation energies were calculated from the slope of the trend line obtained by plotting the logarithm of the specific activity vs. the inverse of the temperature.

**Table 3:** Effect of metal ions on the LAP activity of TmPep1050 and CelM.

Metal ion	TmPep1050 sp act	CelM sp act
-	9.5±0.3	3.8±1.3
Co^2+^	177.6±9.3	319.6±20.8
Ni^2+^	8.0±1.0	8.5±0.6
Zn^2+^	n.d.	1.0±0.1
Mg^2+^	3.9±0.4	3.9±0.3
Cu^2+^	7.9±0.8	4.3±0.4
Ca^2+^	10.6±1.2	2.9±0.2
Mn^2+^	5.7±0.5	4.5±0.4
Fe^2+^	n.d.	4.5±0.6

Specific activities (sp act) are expressed in µmol of p-nitroaniline produced by hydrolysis of the amino acid-pNA derivative per minute and per µmol of enzyme. Enzymatic reactions were carried out at optimal pH (6.6 for TmPep1050 and 6.8 for CelM) and at a metal ion concentration of 100 µM. n.d. not detectable, measured activity below 0.5 µmol min^−1^ µmol^−1^.

Aminoacylase (EC 3.5.1.14) activity was assayed as previously described [30]. Reactions were carried out in 50 mM MOPS buffer pH 7.0 at 60°C with 0.5 mM *N*-acetyl-L-Leu-pNA as substrate. 25 µg of recombinant enzyme was used in each assay.

**Table 4:** Determination of K_m_ and k_cat_ of CelM and TmPep1050.

Enzymes		TmPep1050			CelM	
Substrates	K_m_ (µM)	k_cat_ (s^−1^)	k_cat_/K_m_ (s^−1^ M^−1^)	K_m_ (µM)	k_cat_ (s^−1^)	k_cat_/K_m_ (s^−1^ M^−1^)
L-Leu-pNA	4000±300	0.253±0.019	63.2	3500±400	0.400±0.030	114.3
L-Ile-pNA	1900±200	0.059±0.008	31.1	2800±200	0.110±0.010	39.3
L-Met-pNA	650±50	0.009±0.001	13.8	2900±150	0.072±0.006	24.8

Kinetic parameters for hydrolysis of L-Leu-pNA, L-Ile-pNA, and L-Met-pNA by CelM and TmPep1050. K_m_ and k_cat_ were determined as described in the Materials & Methods section, on the basis of three independent experiments. They are and expressed with SEM. The k_cat_/K_m_ ratio depicts the catalytic efficiency.

### Multiple Sequence Alignments and Protein Pattern Scan

Protein sequences were aligned with T-Coffee using the BLOSUM protein weight matrix [31]. PROSITE was used to retrieve sequences of M42 aminopeptidases and TRI from known proteomes with user-defined protein patterns [32]. The collection of M42 aminopeptidases was built by scanning the UniProtKB/TrEMBL protein database (size filter set at ≥300 and ≤400 residues) with the profile {G-X(15,25)-H-x-[DNE]-X(15,25)-G-X(40,60)-[DEN]-X(30,60)-[DE]-[DNE]-[RKQ]-X(20,40)-E-E-X-[GNASD]-X(20,40)-G-X(50,70)-H}. The distribution of TRI was determined by scanning the UniProtKB/TrEMBL protein database and the PDB (size filter set at ≥750 residues) with the profile {G-S-X-G-D-X(15,25)-R-T-W-G-G}.

## Results

### TmPep1050 and CelM Share Sequence Identity with M42 Aminopeptidases

TmPep1050 from *T. maritima* is assigned to the M42 aminopeptidase family by both CDD (e-value 2.03 e^−111^) and MEROPS. Its structure was determined and deposited by JCSG (http://www.jcsg.org, pdb code 3ISX). DALI [33] was used to perform a structural similarity search with the coordinates of 3ISX, and the closest match identified was TET2 from *P. horikoshii* (PhTET2) (Z score = 38.2). The two structures match well (Fig. S1). Despite this structural similarity, TmPep1050 has been annotated as an endoglucanase because it is 45% identical to CelM from *C. thermocellum*.

The sequences of TmPep1050 and CelM were aligned with those of eight enzymatically or structurally characterized M42 aminopeptidases (Figure 1). PhTET2 [34] shares sequence identity with both CelM and TmPep1050 (48% and 39%, respectively). CelM and TmPep1050 also display five conserved amino acids which are ligands of divalent metal ions (Figure 1). In addition, all of the aligned sequences contain the strictly conserved catalytic glutamic acid (E212 of PhTET2), proposed to act as a general base in hydrolytic catalysis [9], [34], [35].

The CelM sequence was also compared with those of other glycoside hydrolases. It was found to show no similarity to any of the non-redundant sequences of carbohydrate-active enzymes referenced in the CAZy database [36].

### Recombinant TmPep1050 and Dodecameric CelM Display Aminopeptidase Activity

Recombinant TmPep1050 and CelM were produced in *E. coli* and purified by IMAC and gel filtration. At the final step, TmPep1050 eluted at 70±6 kDa, probably as a dimer (predicted molecular weight of the monomer: 37.5 kDa) while two peaks were observed for CelM, one at 407±34 kDa and one at 75±6 kDa. These could correspond, respectively, to a dodecamer and a dimer (predicted molecular weight of the monomer: 36.2 kDa).

The catalytic activities of TmPep1050 and CelM were investigated in the following assays. Firstly, cellulase activity was determined by measuring hydrolysis of cellobiose, CMC, and cellulose substrates, as is usual for cellulases [28]. Under our experimental conditions, TmPep1050 and CelM showed no significant cellulase activity (Table 1).

Secondly, carboxypeptidase assays were carried out with three *N*-terminally blocked peptides. Neither TmPep1050 nor CelM displayed any significant activity (<0.0006 µmol min^−1^ mg^−1^), whereas the positive control, a carboxypeptidase from *Sulfolobus solfataricus,* displayed a specific activity of 0.597±0.138 µmol min^−1^ mg^−1^ with Z-Gly-Tyr, 0.087±0.011 µmol min^−1^ mg^−1^ with Z-Ala-Glu, and 0.319±0.028 µmol min^−1^ mg^−1^ with Z-Gly-Gly-Leu.

Next the aminopeptidase activities of TmPep1050 and CelM were assayed with various L-amino acid-pNA derivatives. The enzymes displayed very similar substrate specificity, preferentially hydrolyzing nonpolar aliphatic L-amino acid-pNA substrates, especially L-leucine-pNA (Table 2). The dodecameric form of CelM showed significant activity (291.7 µmol min^−1^ µmol^−1^), but the dimer proved barely active (6.4 µmol min^−1^ µmol^−1^). Both enzymes were unable to deblock *N*-acetylated-L-Leu-pNA, and thus displayed no aminoacylase activity (specific activity <0.013 µmol min^−1^ mg^−1^). In the presence of the chelating agent EDTA, TmPep1050 and CelM showed more than 98% inhibition (see Table 2). They were also inhibited by bestatin, a specific inhibitor of metalloaminopeptidases [37], with an apparent K_i_ of 292±66 nM for CelM and 432±72 nM for TmPep1050.

### Influence of pH, Temperature, and Metal Ions on the Leucine Aminopeptidase Activity of TmPep1050 and Dodecameric CelM

The leucine aminopeptidase (LAP) activity of TmPep1050 was maximal between pH 6.6 and pH 7.2, rapidly decreasing at pH values below 6.0 and above 7.8 (Figure 2A). CelM likewise showed maximum LAP activity between pH 6.7 and pH 7.1, being inhibited at both acidic and basic pH (Figure 2B). TmPep1050 displayed LAP activity up to 90°C, with a maximum between 70°C and 85°C (Figure 3A). CelM showed maximal LAP activity at around 65°C, with a sharp decrease from about 75°C upward (Figure 3A). The activation energy calculated from the Arrhenius equation fitted to the exponential part of the activity-vs.-temperature plot was 54.62±1.19 kJ mol^−1^ for TmPep1050 and 60.47±2.31 kJ mol^−1^ for CelM (Figure 3B). Different divalent metal ions have been found to influence diversely the specific activity of dinuclear aminopeptidases such as the bovine lens aminopeptidase, the aminopeptidase of *Vibrio proteolyticus* (formerly *Aeromonas proteolytica*), and the M17 leucine aminopeptidase of *Plasmodium falciparum* [38]–[40]. In the case of the M42 aminopeptidases, only Zn^2+^ and Co^2+^ have been found to have a catalytic function [18], [20], [34], [35]. The LAP activity of TmPep1050 and CelM was determined in the presence of various divalent metal ions (Table 3). Both enzymes showed a significant LAP activity increase in the presence of Co^2+^ only. In each case the recorded activity was at least 20 times as high as in the absence of any divalent metal ion or in the presence of any ion other than Co^2+^.

### Kinetic Parameters K_m_ and k_cat_ of TmPep1050 and Dodecameric CelM for Three L-amino Acid-pNA Substrates

Both TmPep1050 and CelM exhibited LAP activity *in vitro* and were able to hydrolyze other nonpolar aliphatic L-amino acid-pNA substrates to a lesser degree. The substrate specificity of each enzyme was studied more closely by determining the k_cat_ and the catalytic efficiency in the presence of L-Leu-pNA, L-Ile-pNA, or L-Met-pNA (Table 4). The substrate saturation curves followed Michaelis-Menten kinetics. Both enzymes showed a clear ‘preference’ for L-Leu-pNA (k_cat_/K_m TmPep1050_ = 63.2 s^−1^ M^−1^; k_cat_/K_m CelM_ = 114.3 s^−1^ M^−1^), as compared to L-Ile-pNA (k_cat_/K_m TmPep1050_ = 31.1 s^−1^ M^−1^; k_cat_/K_m CelM_ = 39.3 s^−1^ M^−1^) and L-Met-pNA (k_cat_/K_m TmPep1050_ = 13.8 s^−1^ M^−1^; k_cat_/K_m CelM_ = 24.8 s^−1^ M^−1^). At their respective optimal temperatures and pH values, CelM and TmPep1050 displayed similar catalytic efficiencies.

### Distribution of M42 Aminopeptidases among the Proteomes of Archaea and Bacteria

Our characterization of CelM as an aminopeptidase led us to believe that several M42 aminopeptidases are annotated wrongly and that the M42 aminopeptidase family is probably incorrectly defined. We therefore undertook to reassess the distribution of M42 aminopeptidases among prokaryotes, ignoring the ‘cellulase’ annotation. On the basis of the conserved amino acids in M42 aminopeptidases according to the Pfam database (PF05343) and of our sequence alignment of characterized M42 aminopeptidases (Figure 1), we defined a sequence pattern for retrieving sequences of potential M42 aminopeptidases. A PROSITE scan with this motif against the UniProtKB/TrEMBL protein database found nearly 3,100 matches. Among these, we rejected the sequences of proteins related to bacterial molybdenum cofactor biosynthesis protein A and eukaryotic inhibitors of protein phosphatase 1. There remained about 2,900 sequences corresponding to proteins classified as aminopeptidases or endoglucanases (Supplementary data). Among these sequences, we focused on those from archaea and bacteria whose complete genomes have been deposited in the European Nucleotide Archive (EMBL database). Among the Archaea, M42 aminopeptidases are found mainly in the phylum Euryarchaeota (except the classes Methanomicrobia and Thermoplasmata) and in the order Thermoproteales (Figure S2). Among the Bacteria, M42 aminopeptidases occur principally in the phyla Thermotogae and Tenericutes, the classes Bacillales and Deionococci, and the order Clostridiaceae (Figure S2). M42 aminopeptidases are sparsely distributed in other phyla, such as the phylum Bacteroidetes and the class γ-Proteobacteria.

Previously, on the basis of studies on TET, Durà *et al.* have suggested that M42 proteins assemble into oligomeric complexes [17]. On the other hand, it has been hypothesized that microorganisms possess either TRI or TET [9]. We therefore compared our proposed distribution of M42 aminopeptidases among archaea and bacteria with that of tricorn peptidases. Although only two prokaryotic tricorn peptidases, those of the archaeon *Thermoplasma acidophilum* and of the bacterium *Streptomyces coelicolor*, have been characterized biochemically or structurally [10], [16] we were able to define a well-conserved profile G-S-X-G-D-X(15,25)-R-T-W-G-G on the basis of nearly 30 occurrences of TRI in archaea and bacteria found in the MEROPS database. A PROSITE scan against this profile was done in the UniProtKB/TrEMBL database, and only 80 sequences were retrieved. Several groups of microorganisms that do not possess TET were found to have TRI, such as the orders Sulfolobales and Thermoplasmatales among the Archaea and the genus Arthrobacter and the family Streptimycetaceae among the Bacteria. Surprisingly, many bacteria seem to possess neither M42 aminopeptidase nor TRI. Moreover, several species may have both TRI and TET, for example *Pyrobaculum aerophilum*, *Petrotoga mobilis*, *Muricauda ruestringensis*, and *Cellulophaga algicola*.

## Discussion

CelM of *C. thermocellum* was previously described as an endoglucanase on the basis of CMCase assays, viscometry measurements, and observed similarities between its amino acid sequence and small parts of CelC and CelH, two components of the cellulosome [22]. Kobayashi *et al.*, however, pointed out that CelM lacks two features commonly found in other cellulases of *C. thermocellum*: Trp residues and repeated sequences [41], [42]. Therefore CelM was viewed as a new type of clostridial endoglucanase. Since then, however, several CelM homologs (PepA of *Lactococcus lactis* [43], PhTET1 of *P. horikoshii* [24], CelM of a *Cytophaga*-like bacterium [26], and three aminopeptidases of *Symbiobacterium thermophilum* [25]) have been characterized, and all of them appear to be M42 aminopeptidases. Cottrell *et al.* also attempted to show that CelM has aminopeptidase activity, but failed to measure any significant activity with glutamine-pNA as substrate and Zn^2+^ as cofactor [26].

The possibility that CelM might be an aminopeptidase rather than an endoglucanase has led us to characterize its enzymatic activity extensively, along with that of TmPep1050, a CelM homolog found in *T. maritima* and annotated as an endoglucanase. Both enzymes emerge as leucine aminopeptidases. Both share sequence identity with PhTET2, a well-characterized M42 aminopeptidase, and TmPep1050 is a structural homolog of PhTET2. Under our experimental conditions (10 µg purified enzyme, a 30-min incubation), CelM and TmPep1050 show no significant cellulase activity towards the substrates cellobiose, CMC, or Whatman filter paper. On the other hand, they both display LAP activity (188.9 and 291.7 µmol min^−1^ µmol^−1^ of enzyme, respectively). By comparison, the CMCase activity of CelM determined by Kobayashi *et al.* was 1.3 µmol min^−1^ µmol^−1^ of enzyme, after an incubation time of 5 hours at 60°C [22]. We can suspect that such activity is not significant in comparison with Cel9I from *C. thermocellum* (CMCase activity of 1,200 µmol min^−1^ µmol^−1^ of enzyme) [44]. Our results show that CelM degrades nonpolar aliphatic L-amino acid-pNA substrates and that Co^2+^ is required for maximal activity *in vitro*. These findings explain why no aminopeptidase activity was observed previously for CelM [26]. Furthermore, the measured specific activities for TmPep1050 and CelM are in the same range (µmol min^−1^ µmol^−1^ of enzyme) as those of other characterized M42 aminopeptidases [19], [25], [34], and the estimated K_m_ values are close to those observed for YsdC and PhTET2 [45].

Our characterization of CelM has led us to view CelM homologs as aminopeptidases in our database search for M42 aminopeptidases in archaea and bacteria. We have found this protein family to be widely represented in both kingdoms, but representatives are scarce to absent in some phyla, such as the Protobacteria. According to the current view of peptide degradation in prokaryotes, each organism should possess either a TET aminopeptidase or a TRI peptidase [9]. However, TRI seems to be present in only a small set of prokaryotes, and many organisms lack both TRI and TET. As few bacterial TRI peptidases have been characterized, our determination of their distribution could be somewhat biased, but our results are in accordance with a previous study on tricorn-like proteases in bacteria [15]. Unexpectedly, we observe that several organisms share both TET and TRI, in contradiction to the current hypothesis. This finding is in agreement with data available from MEROPS. For instance, *Pyrobaculum aerophilum* possesses both a PhTET1 homolog (MER016947) and a TRI peptidase (MER016957). Perhaps both enzymes are co-produced and participate together in peptide degradation, or perhaps both complexes are maintained to allow regulation in response to stress [46]. The expression of M42 aminopeptidase genes does seem to be regulated, as demonstrated for two TET aminopeptidases of *Thermococcus kodakarensis* whose syntheses respond to heat stress and oxidative stress. Our study strongly suggests that other peptide-degrading complexes may exist, at least in prokaryotes lacking both TET and TRI. These might be aminopeptidases similar to PepA/PepB, PepN, and the thimet oligopeptidase homolog OpdA, extensively studied in *E. coli* and *Salmonella typhimurium* and proposed to act downstream from the proteasome [47]–[50]. Yet according to our results on the distribution of M42 aminopeptidases, *E. coli* also possesses three M42 aminopeptidases. The activity of one of them, YpdE, has been characterized previously [51], and it is found in a dodecameric state (unpublished data). Why this species maintains several complexes capable of degrading peptides has not yet been studied. The fact that the enzymes responsible for peptide degradation in prokaryotes have not been characterized *in vivo* hinders our understanding of the pathways in which they participate.

## Supporting Information

Figure S1 **Structural alignment of 3ISX vs 1XFO.** 1092 atoms were aligned with a root mean square deviation of 1.02 Å. Colored boxes beneath each amino acid of 1XFO represent the spatial deviation between 3ISX and 1XFO, ranging from dark blue (RMS <0.5) to red (RMS >5) through green (RMS = 2.5). * and • display conserved amino acid and homologous residues respectively.
(PDF)

Figure S2 **Distribution of TET aminopeptidases and TRI peptidases among Archaea and Bacteria whose genomes were deposited at in European Nucleotide Archive (EMBL database).** Phylogenetic tree build with NCBI Taxonomy Common Tree. Names of organism possessing TET are in red, TRI in blue, TET and TRI in green.
(PDF)
