# Supplementary material for: Functional Characterization of Two M42 Aminopeptidases Erroneously Annotated as Cellulases
Source: PLoS One. 2012 Nov 30;7(11):e50639. doi: 10.1371/journal.pone.0050639 (PMC3511314; doi:10.1371/journal.pone.0050639)
Supplement: Figure S1 — Structural alignment of 3ISX vs 1XFO. 1092 atoms were aligned with a root mean square deviation of 1.02 Å. Colored boxes beneath each amino acid of 1XFO represent the spatial deviation between 3ISX and 1XFO, ranging from dark blue (RMS <0.5) to red (RMS >5) through green (RMS = 2.5). * and • display conserved amino acid and homologous residues respectively. (PDF) [file pone.0050639.s001.pdf]

```
3ISX     1              MKELIRKLT EAFGPSGREE E-VRSIILEE LEGHIDGHRI
1XFO    -3   RGSHMEVRNM VDYELLKKVV EAPGVSGYEF LGIRDVVIEE IKDYVDEVKV
                           **●●*  ** * ** *    ●* ●●●** ●   ●*  ●●

3ISX    39   DGLGNLIVWK GSGEKKVILD AHIDEIGVVV TNVDDKGFLT IEPVGGVSPY
1XFO    47   DKLGNVIAHK KGEGPKVMIA AHMDQIGLMV THIEKNGFLR VAPIGGVDPK
             * ***●*  *  ●   **●●  **●*●**●●* *●●● ●***  ● *●*** * 

3ISX    90   MLLGKRIRFE N----GTIGV VGMEGETTEE RQENVRKLSF DKLFIDIGAN
1XFO    97   TLIAQRFKVW IDKGKFIYGV GASVP----- ------APDW DQIFIDIGAE
              *●● *●●           **  ●                    * ●******●

3ISX   135   SREEAQK-MC PIGSFGVYDS GFVEVSG-KY VSKAMDDRIG CAVIVEVFKR
1XFO   147   SKEEAEDMGV KIGTVITWDG RLERLGKHRF VSIAFDDRIA VYTILEVAKQ
             *●***●      **●    *●  ●  ●●  ●● ** * ****●    *●** *●

3ISX   183   IK-PAVTLYG VFSVQEEVGL VGASVAGYGV PADEAIAIDV TDSADTPKAI
1XFO   197   LKDAKADVYF VATVQEEVGL RGARTSAFGI EPDYGFAIDV TIAADIPGTP
             ●* ●   ●*  * ●*******  **  ●●●*●  ●* ●●**** * ●** * ● 

3ISX   232   KR-HAMRLSG GPALKVKDRA SISSKRILEN LIEIAEKFDI KYQMEVLTFG
1XFO   247   EHKQVTHLGK GTAIKIMDRS VICHPTIVRW LEELAKKHEI PYQLEILLGG
              ● ●  ●*●  * *●*●●**●  *    *●   * *●* * ●*  ** *●*  *

3ISX   281   GTNAMGYQRT REGIPSATVS IPTRYVHSPS EMIAPDDVEA TVDLLIRYLG
1XFO   297   GTDAGAIHLT KAGVPTGALS VPARYIHSNT EVVDERDVDA TVELMTKALE
             **●* ● ● * ● *●*●●●●* ●*●**●** ● * ●   **●* **●*● ● * 

3ISX   331   A -------- ---------- ---------- ---------- ----------
1XFO   347   NIHELKI-
```

Figure S1 Structural alignment of 3ISX vs 1XFO. 1092 atoms were aligned with a root mean square deviation of 1.02 Å. Colored boxes beneath each amino acid of 1XFO represent the spatial deviation between 3ISX and 1XFO, ranging from dark blue (RMS < 0.5) to red (RMS > 5) through green (RMS = 2.5). * and • display conserved amino acid and homologous residues respectively.
