# Supplementary material for: Functional Characterization of Two M42 Aminopeptidases Erroneously Annotated as Cellulases
Source: PLoS One. 2012 Nov 30;7(11):e50639. doi: 10.1371/journal.pone.0050639 (PMC3511314; doi:10.1371/journal.pone.0050639)
Supplement: Figure S2 — Distribution of TET aminopeptidases and TRI peptidases among Archaea and Bacteria whose genomes were deposited at in European Nucleotide Archive (EMBL database). Phylogenetic tree build with NCBI Taxonomy Common Tree. Names of organism possessing TET are in red, TRI in blue, TET and TRI in green. (PDF) [file pone.0050639.s002.pdf]

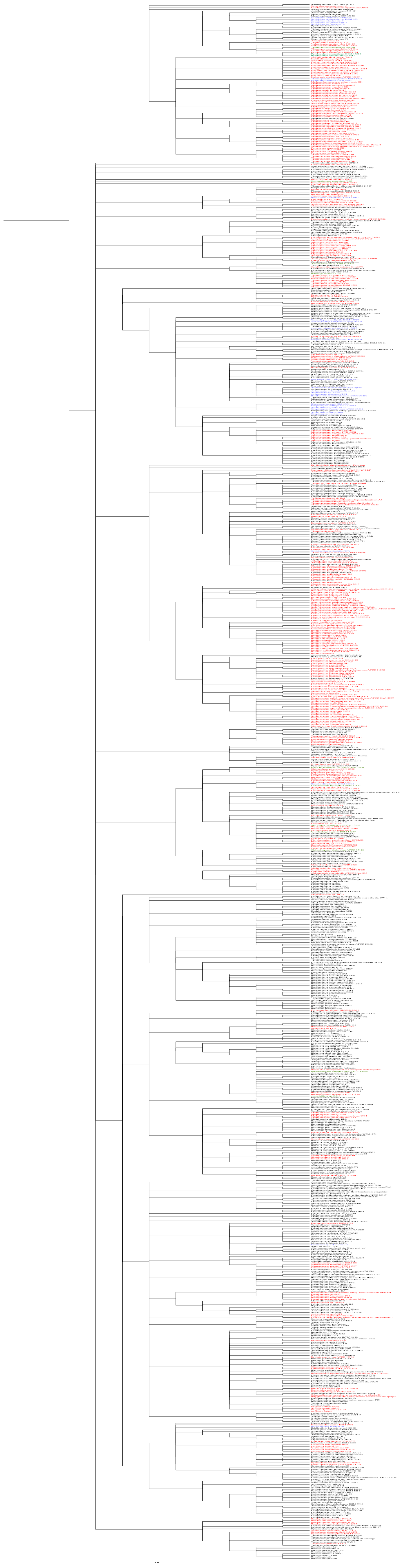

Figure S2 Distribution of TET aminopeptidases and TRI peptidases among Archaea and Bacteria whose genomes were deposited at in European Nucleotide Archive (EMBL database). Phylogenetic tree build with NCBI Taxonomy Common Tree. Names of organism possessing TET are in red, TRI in blue, TET and TRI in green.
